# Application of a methicillin-resistant *Staphylococcus aureus* risk score for community-onset pneumonia patients and outcomes with initial treatment

**DOI:** 10.1186/s12879-015-1119-1

**Published:** 2015-09-18

**Authors:** Besu F. Teshome, Grace C. Lee, Kelly R. Reveles, Russell T. Attridge, Jim Koeller, Chen-pin Wang, Eric M. Mortensen, Christopher R. Frei

**Affiliations:** St. Louis College of Pharmacy, St. Louis, MO USA; Pharmacotherapy Division, College of Pharmacy, The University of Texas at Austin, Austin, TX USA; Pharmacotherapy Education and Research Center, School of Medicine, The University of Texas Health Science Center at San Antonio, 7703 Floyd Curl Dr., MSC-6220, San Antonio, TX 78229 USA; Feik School of Pharmacy, University of the Incarnate Word, San Antonio, TX USA; South Texas Veterans Health Care System, San Antonio, TX USA; Department of Epidemiology and Biostatistics, University of Texas Health Science Center, San Antonio, TX USA; The VA North Texas Health Care System, Dallas, TX USA; The University of Texas Southwestern Medical Center, Dallas, TX USA

## Abstract

**Background:**

Community-onset (CO) methicillin-resistant *Staphylococcus aureus* (MRSA) pneumonia is an evolving problem, and there is a great need for a reliable method to assess MRSA risk at hospital admission. A new MRSA prediction score classifies CO-pneumonia patients into low, medium, and high-risk groups based on objective criteria available at baseline. Our objective was to assess the effect of initial MRSA therapy on mortality in these three risk groups.

**Methods:**

We conducted a retrospective cohort study using data from the Veterans Health Administration (VHA). Patients were included if they were hospitalized with pneumonia and received antibiotics within the first 48 h of admission. They were stratified into MRSA therapy and no MRSA therapy treatment arms based on antibiotics received in the first 48 h. Multivariable logistic regression was used to adjust for potential confounders.

**Results:**

A total of 80,330 patients met inclusion criteria, of which 36 % received MRSA therapy and 64 % did not receive MRSA therapy. The majority of patients were classified as either low (51 %) or medium (47 %) risk, with only 2 % classified as high-risk. Multivariable logistic regression analysis demonstrated that initial MRSA therapy was associated with a lower 30-day mortality in the high-risk group (adjusted odds ratio 0.57; 95 % confidence interval 0.42–0.77). Initial MRSA therapy was not beneficial in the low or medium-risk groups.

**Conclusions:**

This study demonstrated improved survival with initial MRSA therapy in high-risk CO-pneumonia patients. The MRSA risk score might help spare MRSA therapy for only those patients who are likely to benefit.

**Electronic supplementary material:**

The online version of this article (doi:10.1186/s12879-015-1119-1) contains supplementary material, which is available to authorized users.

## Background

Pneumonia is a major cause of mortality in the United States, with a reported 49,597 deaths in 2010 [1]. Community-onset (CO) pneumonia is defined as pneumonia that occurs in the community and up to 48 h into hospital admission. It encompasses both community-acquired pneumonia (CAP) and healthcare-associated pneumonia (HCAP). Importantly, HCAP patients are at increased risk of methicillin-resistant *Staphylococcus aureus* (MRSA) pneumonia [2–4]. Lastly, MRSA pneumonia is associated with greater morbidity and mortality than pneumonia caused by other etiologies, possibly due to the virulent and resistant nature of the MRSA pathogen [4]. MRSA accounts for 20–40 % of pneumonia cases that occur after 48 h into hospital admission and 2–25 % of CO cases overall [4–8].

Previous studies have demonstrated that rapid initiation of appropriate antibiotic therapy is associated with improved survival in hospitalized patients with infections [9, 10]; therefore, there is a great need for a reliable method to assess CO-MRSA pneumonia risk at admission. Guidelines recommend use of the HCAP criteria to determine need for empiric MRSA therapy, but this definition lacks specificity for CO-MRSA pneumonia and may lead to overuse of broad-spectrum antibiotic therapy [2, 11]. Finally, prior studies have demonstrated that when HCAP patients received guideline-recommended, broad-spectrum therapy (including MRSA therapy), outcomes were no better than when similar patients received alternative antibiotics [12, 13].

Guidance is needed for clinicians to identify those CO-pneumonia patients who might benefit from empiric MRSA therapy.

Recently, Shorr *et al.* derived a clinical prediction score that stratified patients with CO-pneumonia by their MRSA risk [14]. The risk score consisted of eight variables. Two points were assigned for recent hospitalization or intensive care unit (ICU) admission and one point was assigned for each of the following: age <30 or >79 years, prior intravenous (IV) antibiotics in last 30 days, dementia, cardiovascular disease, female with diabetes, or recent exposure to a nursing home, long-term care facility, or skilled nursing facility. The total score ranged from 0 to 10, and patients were stratified into low (0–1), medium (2–5), and high (6–10) risk groups. The CO-MRSA pneumonia prevalence increased from <10 % in the low-risk group to >30 % in the high-risk group. The authors concluded that this risk score could help identify those patients at low risk of MRSA, for which MRSA therapy could be spared. They postulated that patients in the high-risk group might benefit from MRSA therapy [14]; however, this has yet to be proven.

The new MRSA risk score could help guide empiric MRSA therapy; however, studies are needed to determine which, if any, of the MRSA risk groups benefit from such therapy. Our primary objective was to compare the effect of MRSA therapy on 30-day patient mortality among CO-pneumonia patients in the three MRSA risk groups (low, medium, and high-risk). Our secondary objective was to determine the association of MRSA risk score with 30-day mortality.

## Methods

This study used administrative data from the Veterans Health Administration (VHA) database. Description of the methods used to build this database have been previously reported [12, 15, 16]. In brief, we performed a retrospective, population-based cohort study using administrative data from the VHA system between fiscal years 2002 and 2007. These data are from over 150 VHA hospitals and 850 VHA outpatient clinics. Data for this study were obtained from the VHA electronic medical record system that includes administrative, clinical, laboratory, and pharmacy data. The Institutional Review Board of the University of Texas Health Science Center at San Antonio and the South Texas Veterans Health Care System Research and Development committee approved this study.

Patients were included if they were ≥65 years of age and had either a primary discharge diagnosis of pneumonia/influenza (International Classification of Diseases, Ninth Revision, Clinical Modification [ICD-9-CM] codes 480.0–483.99 or 485–487) (Additional file 1) or a secondary discharge diagnosis of pneumonia/influenza plus a primary diagnosis of respiratory failure (ICD-9-CM code 518.81), or sepsis (ICD-9-CM code 038.xx) in fiscal years 2002–2007. If a patient was admitted more than once during the study period, only the first hospitalization was included. Patients were excluded if they did not receive antimicrobial therapy within the first 48 h of admission.

Baseline demographics were recorded at the time of admission. Antibiotic use was recorded for the first 48 h of admission. Comorbid conditions were determined using ICD-9-CM codes from outpatient and inpatient care in accordance with the Charlson comorbidity scoring system [17, 18]. Organ failure included acute and chronic conditions (Additional file 1).

The MRSA risk score variables were defined as: patient age >79 years, hospitalization in the past 90 days, (ICU) admission, outpatient (IV) antibiotic therapy within the past 90 days, nursing home resident in the last 90 days, cerebrovascular disease, dementia, and female with diabetes mellitus. These were based on the MRSA risk score developed by Shorr *et al.* and modified for our database. All of our patients were ≥65 years of age, so the criteria related to age <30 years did not apply. Also, recent hospitalization was not limited to stays of ≥2 days, prior IV antibiotic therapy was extended from 30 to 90 days, and ICU admission was not limited to on or before index culture [14]. Each variable contributed one point to the risk score, except for hospitalization in the past 90 days and ICU admission, for which each contributed two points. Patients were stratified into three risk groups based on their risk score: low (0–1), medium (2–5), and high (6–10) [14].

Patients who met study criteria were divided into two groups: the “MRSA therapy” group and the “no MRSA therapy” group. Initial MRSA therapy was defined as the receipt of either vancomycin or linezolid within the first 48 hours of admission. Patients were also categorized based on the receipt of guideline-concordant community-acquired pneumonia (GC-CAP) therapy [19], pseudomonal therapy, and atypical therapy (Table 1). Pneumonia pathogens, including MRSA, were identified using ICD-9-CM codes (Additional file 1).

**Table 1:** Antibiotic therapy definitions

MRSA therapy	
• Vancomycin	
• Linezolid	
Guideline-Concordant CAP therapy	
Ward patients	ICU patients
• Beta-lactam^a^ *plus* (macrolide^b^ or doxycycline)	• Beta-lactam^a^ *plus* (macrolide^b^ or doxycycline)
• Respiratory fluoroquinolone^c^	• Beta-lactam^a^ *plus* respiratory fluoroquinolone^c^
Pseudomonal therapy	
• Antipseudomonal beta-lactam^d^ *plus* antipseudomonal fluoroquinolone^e^	
• Antipseudomonal beta-lactam^d^ *plus* aminoglycoside^f^	
Atypical therapy	
• Macrolide^b^	
• Doxycycline	
• Any fluoroquinolone	

*CAP* Community-acquired pneumonia, *ICU* Intensive care unit

^a^Beta-lactam includes cefotaxime, ceftriaxone, ampicillin-sulbactam, ertapenum, or aztreonam

^b^Macrolide includes azithromycin, clarithromycin, or erythromycin

^c^Respiratory fluoroquinolone includes moxifloxacin, levofloxacin, or gatifloxacin

^d^Antipseudomonal beta-lactam includes cefepime, ceftazidime, imipenem-cilastatin, meropenem, piperacillin-tazobactam, or ticarcillin-clavulanate, aztreonam

^e^Antipseudomonal fluoroquinolone includes ciprofloxacin or levofloxacin

^f^Aminoglycoside includes gentamicin, tobramycin, or amikacin

All-cause 30-day patient mortality was the primary study outcome. Previous research has demonstrated that 30-day mortality is more closely associated with pneumonia-related mortality, as compared to 60-day or 90-day mortality [20]. Mortality was assessed using the VHA vital status file, which has been demonstrated to have 98 % exact agreement with the National Death Index, the “gold standard” to determine mortality [21].

### Statistical analyses

All statistical analyses were conducted using JMP 10.0® (SAS Corp, Cary, NC). Chi-square or Fisher’s exact test were used to compare categorical variables between study arms (Table 2). Continuous variables were compared using the Wilcoxon Rank Sum test (Table 2). For bivariate statistical tests, we defined significance as a two-tailed alpha ≤0.0001 to avoid spurious associations in this large patient cohort.

**Table 2:** Baseline characteristics grouped by MRSA therapy

	Overall (*n* = 80,330)	MRSA therapy (*n* = 29,254)	No MRSA therapy (*n* = 51,076)	*P*-value^*^
Patient age (years), median (IQR)	78 (72–83)	78 (73–83)	77 (72–83)	0.1016
Male, %	98.3	98.3	98.3	0.6847
Race, %				
White	81.1	79.1	82.3	< 0.0001
Black	13.1	15.4	11.7	< 0.0001
Other	5.8	5.5	6.0	< 0.0001
Hispanic ethnicity, %	7.0	8.0	6.4	< 0.0001
MRSA risk score variables, % (1 point, unless noted)				
Age >79	43.8	44.0	43.7	0.3867
Hospitalization in the past 90 days (2 points)	27.8	33.7	24.5	< 0.0001
Intensive care unit admission (2 points)	21.1	29.3	16.4	< 0.0001
Outpatient IV antibiotic therapy in past 90 days	4.9	5.3	4.7	0.0001
Nursing home resident in last 90 days	1.0	1.1	0.9	0.0028
Cerebrovascular disease	18.1	19.6	17.3	< 0.0001
Dementia	5.2	5.8	4.9	< 0.0001
Female with diabetes mellitus	0.4	0.4	0.4	0.8129
MRSA risk score, median (IQR)	1 (0–3)	2 (1–3)	1 (0–2)	< 0.0001
Low (0–1), %	51.4	41.6	57.0	< 0.0001
Medium (2–5), %	47.3	56.4	42.1	< 0.0001
High (6–10), %	1.3	2.1	0.9	< 0.0001
Charlson comorbidity score, median (IQR)	2 (1–4)	3 (1–4)	2 (1–4)	< 0.0001
Comorbid conditions, %				
Myocardial infarction	7.2	7.6	7.0	0.0020
Heart failure	25.9	27.0	25.3	< 0.0001
Chronic obstructive pulmonary disease	48.7	45.5	50.5	< 0.0001
Liver disease	1.3	1.5	1.2	0.0035
Renal disease	14.1	17.2	12.3	< 0.0001
Diabetes	30.5	31.8	29.8	< 0.0001
Neoplastic disease	25.2	26.2	24.7	< 0.0001
HIV/AIDS	0.2	0.3	0.2	0.0961
Medication use within 90 days, %				
Cardiovascular medications	66.5	64.9	67.3	< 0.0001
Anti-diabetic medications	22.2	22.6	22.1	0.0852
Inhaled corticosteroids	21.1	18.9	22.4	< 0.0001
Systemic corticosteroids ^a^	22.2	21.8	22.5	0.0217
Pulmonary medications	34.8	31.4	36.8	< 0.0001
Vasopressors, %	10.2	15.2	7.2	< 0.0001
Invasive mechanical ventilation, %	11.1	16.3	8.2	< 0.0001
Noninvasive mechanical ventilation, %	4.0	5.7	3.1	< 0.0001
Hemodialysis, %	18.3	22.7	15.7	< 0.0001
Organ failure, %				
Any organ failure, %	32.2	41.8	26.7	< 0.0001
Respiratory	14.4	19.4	11.5	< 0.0001
Cardiovascular	9.7	13.0	7.8	< 0.0001
Neurological	2.5	3.3	2.0	< 0.0001
Renal	20.1	26.8	16.2	< 0.0001
Hematologic	4.1	5.5	3.3	< 0.0001
Hepatic	0.7	0.9	0.6	< 0.0001
Antibiotic therapy, %				
Guideline-concordant CAP therapy	64.1	64.9	63.6	0.0001
Pseudomonal therapy	17.5	31.1	9.7	< 0.0001
Atypical therapy	75.2	83.6	70.5	< 0.0001

*MRSA* Methicillin-resistant *Staphylococcus aureus*, *IQR* Interquartile range, *IV* Intravenous, *HIV/AIDS* Human immunodeficiency virus/acquired immunodeficiency syndrome, *CAP* Community-acquired pneumonia

^a^ Includes oral and/or injectable corticosteroids

^*^ Comparison between “MRSA therapy” versus “no MRSA therapy” groups

Separate multivariable logistic regression models were constructed to examine if MRSA therapy was associated with 30-day mortality in the overall population and each of the three MRSA risk groups. The dependent variable was 30-day patient mortality, and the independent variable was MRSA therapy versus no MRSA therapy. Covariates included all unbalanced characteristics that were significant in the bivariate analysis comparing 30-day mortality versus no 30-day mortality (Additional file 2). Finally, MRSA culture-positivity was also entered into the model. Adjusted odds ratios (aORs) and 95 % confidence intervals (95 % CIs) were calculated; those 95 % CIs that did not cross one were considered to be statistically significant.

A few of the variables were excluded from the model because of collinearity. Collinearity was determined through theoretical relations for select variables. For instance, most patients on hemodialysis also had renal failure; therefore, hemodialysis was chosen for the model. Likewise, most patients who had diabetes mellitus also received anti-diabetic medications; therefore, the anti-diabetic medications variable was chosen for the model. The Charlson score and the “any organ failure” variables were excluded from the model because individual comorbidities and organ failures were already included in the model. Individual MRSA risk score variables were also excluded from the model because we ran separate multivariable models for the three risk groups, and these individual characteristics were used to define those risk groups.

The final list of 25 covariates included: patient age, race, Hispanic ethnicity, myocardial infarction, heart failure, chronic obstructive pulmonary disease (COPD), liver disease, renal disease, neoplastic disease, cardiovascular medications, anti-diabetic medications, inhaled corticosteroids, pulmonary medications, vasopressors, invasive and non-invasive mechanical ventilation, respiratory failure, cardiovascular failure, neurological failure, renal failure, hematological failure, hepatic failure, GC-CAP therapy, pseudomonal therapy, atypical therapy, and MRSA culture-positivity.

## Results

### Overall population

Baseline patient characteristics are shown in Table 2. A total of 80,330 patients met inclusion criteria, with 36 % in the MRSA therapy group and 64 % in the no MRSA therapy group. Patients were predominately elderly (median age 78 years), white (81 %) men (98 %). Age >79 was the most common MRSA risk factor (44 %), followed by hospitalization in the past 90 days (28 %) and ICU admission (21 %). There were very few women with diabetes mellitus (0.4 %) in this population. The median MRSA risk score was 1 (interquartile range [IQR] 0–3). The majority of patients were classified as either low (51 %) or medium (47 %) risk, with only 2 % classified as high-risk. None of the patients scored above an 8 on the MRSA risk score.

The median (IQR) Charlson score was 2 (1–4), and common comorbidities included COPD (49 %), diabetes (31 %), heart failure (26 %), and neoplastic disease (25 %). The most commonly used medications within 90 days prior to admission included cardiovascular medications (67 %) and pulmonary medications (35 %). Organ failure occurred in 32 % of patients. Finally, most patients received atypical (75 %) and GC-CAP (64 %) therapy.

### Baseline characteristics

Patient age and sex were similar between MRSA therapy and no MRSA therapy groups. A lower proportion of white patients received MRSA therapy, while higher proportions of black and Hispanic patients received MRSA therapy.

Charlson scores were higher in the MRSA therapy group and these patients had a higher prevalence of heart failure, renal disease, diabetes, and neoplastic disease. In addition, the MRSA therapy group had a higher prevalence of vasopressor use, mechanical ventilation, hemodialysis, organ failure, GC-CAP therapy, pseudomonal therapy, and atypical therapy. In contrast, the no MRSA therapy group had a higher prevalence of COPD and were more likely to receive cardiovascular medications, inhaled corticosteroids, and pulmonary medications in the last 90 days.

### Bacterial pathogens

The prevalence of bacterial pathogens is shown in Table 3. *Staphylococcus aureus* was the most commonly isolated pathogen and the majority of those isolates were MRSA. *Streptococcus pneumoniae* was the second most common pathogen. The MRSA therapy group had more patients who were culture-positive and had higher rates of *Staphylococcus aureus*, MRSA, *Pseudomonas* spp., and most other gram-negative pathogens, except for *Haemophilus influenzae*.

**Table 3:** Bacterial pathogen distribution grouped by MRSA therapy

	Overall (*n* = 80,330)	MRSA therapy (*n* = 29,254)	No MRSA therapy (*n* = 51,076)	*P*-value^*^
Organism identified, %	10.3	13.9	8.2	< 0.0001
Single organism identified	9.3	12.1	7.7	< 0.0001
Multiple organisms identified	1.0	1.9	0.5	< 0.0001
Gram-positive pathogens, %				
*Streptococcus pneumoniae*	2.8	2.7	2.9	0.0571
*Streptococcus*, other	0.4	0.5	0.4	0.0270
*Staphylococcus aureus*	5.1	8.7	3.1	< 0.0001
MRSA	3.3	5.1	2.3	< 0.0001
Gram-negative pathogens, %				
*Klebsiella pneumoniae*	0.7	1.0	0.6	< 0.0001
*Pseudomonas* spp.	1.5	1.9	1.2	< 0.0001
*Haemophilus influenzae*	0.8	0.5	0.9	< 0.0001
*Escherichia coli*	0.2	0.4	0.2	< 0.0001
Other gram-negatives	0.5	0.6	0.4	0.0007
Atypical pathogens, %				
*Mycoplasma pneumoniae*	< 0.1	< 0.1	0.1	0.0839
*Legionella* spp.	0.1	0.2	0.1	0.2029
*Chlamydia* spp.	< 0.1	<0.1	< 0.1	0.7561
Anaerobes, %	0.1	0.1	0.1	0.0139

*MRSA* Methicillin-resistant *Staphylococcus aureus*

^*^ Comparison between “MRSA therapy” versus “no MRSA therapy” groups

### Patient mortality

**Fig. 1:**
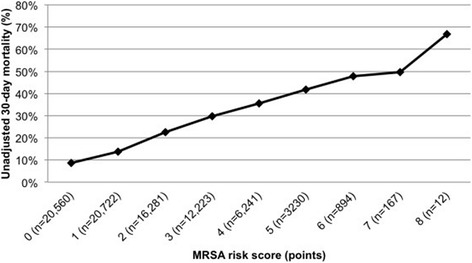
Unadjusted 30-day patient mortality by MRSA risk score. *MRSA* Methicillin-resistant *Staphylococcus aureus*

**Fig. 2:**
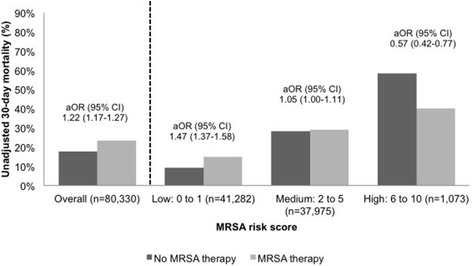
30-day patient mortality by MRSA risk score and MRSA therapy. *aOR* Adjusted Odds Ratio, *95 % CI* 95 % Confidence Interval, *MRSA* Methicillin-resistant *Staphylococcus aureus*

The overall 30-day patient mortality rate was 20 %, and the unadjusted 30-day mortality increased from the low (11 %), medium (27 %), and high (48 %) risk groups (*p* <0.0001). In addition, an increase in unadjusted 30-day mortality was observed with each additional point of the MRSA risk score (Fig. 1). Unadjusted 30-day mortality was higher among patients who received MRSA therapy in the low-risk group (15 % versus 9 %; *p* <0.0001), but lower in the high-risk group (40 % versus 58 %; *p* <0.0001) (Fig. 2). After adjustment for potential confounders, MRSA therapy was associated with higher 30-day mortality in the low-risk group (aOR 1.47; 95 % CI 1.37–1.58) and lower 30-day mortality in the high-risk group (aOR 0.57; 95 % CI 0.42–0.77) (Fig. 2).

More than 40 characteristics were compared among patients who survived and those who died within 30-days of hospital discharge (Table 4). The multivariable regression analysis revealed that several of those variables were independently associated with 30-day mortality (Table 5). Risk factors varied for low, medium, and high-risk groups.

**Table 4:** Baseline characteristics grouped by 30-day patient mortality

	Overall (*n* = 80,330)	30-day mortality (*n* = 15,909)	No 30-day mortality (*n* = 64,421)	*P*-value^*^
Patient age (years), median (IQR)	78 (72–83)	79 (74–84)	77 (72–82)	< 0.0001
Male, %	98.3	98.5	98.2	0.0100
Race, %				
White	81.1	77.3	82.0	< 0.0001
Black	13.1	14.9	12.6	< 0.0001
Other	5.8	7.8	5.3	< 0.0001
Hispanic ethnicity, %	7.0	7.9	6.8	< 0.0001
MRSA risk score variables, % (1 point, unless noted)				
Age >79	43.8	51.0	42.0	< 0.0001
Hospitalization in the past 90 days (2 points)	27.8	40.7	24.7	< 0.0001
Intensive care unit admission (2 points)	21.1	42.0	16.0	< 0.0001
Outpatient IV antibiotic therapy in past 90 days	4.9	6.0	4.7	< 0.0001
Nursing home resident in last 90 days	1.0	0.8	1.0	0.0292
Cerebrovascular disease	18.1	20.0	17.7	< 0.0001
Dementia	5.2	6.7	4.9	< 0.0001
Female with diabetes mellitus	0.4	0.4	0.4	0.8881
MRSA risk score, median (IQR)	1 (0–3)	2 (1–2)	1 (0–2)	< 0.0001
Low (0–1), %	51.4	28.4	57.1	< 0.0001
Medium (2–5), %	47.3	68.4	42.1	< 0.0001
High (6–10), %	1.3	3.25	0.9	< 0.0001
Charlson comorbidity score, median (IQR)	2 (1–4)	3 (1–5)	2 (1–4)	< 0.0001
Comorbid conditions, %				
Myocardial infarction	7.3	8.7	6.9	< 0.0001
Heart failure	25.9	28.2	25.4	< 0.0001
Chronic obstructive pulmonary disease	48.7	41.7	50.4	< 0.0001
Liver disease	1.3	2.3	1.1	< 0.0001
Renal disease	14.1	16.7	13.4	< 0.0001
Diabetes	30.5	30.3	30.5	0.5493
Neoplastic disease	25.2	31.1	23.8	< 0.0001
HIV/AIDS	0.2	0.2	0.2	0.2145
Medication use within 90 days, %				
Cardiovascular medications	66.5	60.0	68.0	<0.0001
Anti-diabetic medications	22.2	20.0	22.8	<0.0001
Inhaled corticosteroids	21.1	14.8	22.7	<0.0001
Systemic corticosteroids^a^	22.2	21.1	22.5	0.0002
Pulmonary medications	34.8	27.8	36.6	< 0.0001
Vasopressors, %	10.2	26.9	6.0	< 0.0001
Invasive mechanical ventilation, %	11.1	26.8	7.3	< 0.0001
Noninvasive mechanical ventilation, %	4.0	7.1	3.3	< 0.0001
Hemodialysis, %	18.3	23.5	17.0	< 0.0001
Organ failure, %				
Any organ failure, %	32.2	57.8	25.9	< 0.0001
Respiratory	14.4	32.6	9.9	< 0.0001
Cardiovascular	9.7	22.1	6.7	< 0.0001
Neurological	2.5	4.2	2.0	< 0.0001
Renal	20.1	35.3	16.3	< 0.0001
Hematologic	4.1	8.3	3.0	< 0.0001
Hepatic	0.7	2.0	0.3	< 0.0001
Antibiotic therapy, %				
MRSA therapy	36.4	42.9	34.8	< 0.0001
Guideline-concordant CAP therapy	64.1	38.6	70.4	< 0.0001
Pseudomonal therapy	17.5	23.8	15.9	< 0.0001
Atypical therapy	75.2	54.3	80.4	< 0.0001

*MRSA* Methicillin-resistant *Staphylococcus aureus*, *IQR* Interquartile range, *IV* Intravenous, *HIV/AIDS* Human immunodeficiency virus/acquired immunodeficiency syndrome, *CAP* Community-acquired pneumonia

^a^Includes oral and/or injectable corticosteroids

^*^Comparison between “30-day mortality” versus “no 30-day mortality” groups

**Table 5:** Risk factors for 30-day patient mortality grouped by MRSA risk score

	Adjusted Odds ratio (95 % confidence interval)			
Risk score	All (*n* = 80,330)	0–1 (*n* = 41,282)	2–5 (*n* = 37,975)	6–10 (*n* = 1,073)
MRSA therapy	**1.22 (1.17-1.27)**	**1.47 (1.37-1.58)**	1.05 (1.00-1.11)	**0.57 (0.42-0.77)**
Age (1-year increments)	**1.05 (1.04-1.05)**	**1.05 (1.05-1.06)**	**1.04 (1.03-1.04)**	**1.05 (1.02-1.08)**
Race	0.99 (0.94-1.05)	0.95 (0.86-1.06)	0.98 (0.91-1.05)	1.18 (0.85-1.64)
Hispanic ethnicity	**0.85 (0.79-0.92)**	**0.84 (0.73-0.96)**	**0.86 (0.79-0.95)**	0.79 (0.47-1.32)
Comorbid conditions				
Myocardial infarction	**1.14 (1.06-1.23)**	1.11 (0.94-1.30)	1.05 (0.97-1.14)	1.06 (0.74-1.51)
Heart failure	**1.16 (1.11-1.21)**	1.07 (0.97-1.17)	**1.11 (1.05-1.18)**	1.16 (0.88-1.53)
COPD	0.96 (0.91-1.00)	0.92 (0.85-1.00)	0.95 (0.89-1.01)	1.30 (0.96-1.77)
Liver disease	**1.79 (1.54-2.08)**	1.34 (0.97-1.87)	**1.80 (1.50-2.14)**	**3.46 (1.08-13.51)**
Renal disease	0.98 (0.93-1.04)	0.91 (0.81-1.02)	0.96 (0.90-1.03)	1.12 (0.83-1.53)
Neoplastic disease	**1.54 (1.47-1.60)**	**1.57 (1.46-1.69)**	**1.48 (1.40-1.56)**	1.18 (0.87-1.59)
Medication use, by class				
Cardiovascular medications	**0.72 (0.69-0.75)**	**0.60 (0.56-0.64)**	**0.78 (0.73-0.82)**	0.91 (0.66-1.24)
Anti-diabetic medications	**0.92 (0.87-0.96)**	**0.78 (0.71-0.86)**	0.95 (0.90-1.01)	0.95 (0.70-1.29)
Inhaled corticosteroids	**0.76 (0.72-0.81)**	**0.73 (0.65-0.81)**	**0.80 (0.74-0.86)**	1.13 (0.77-1.65)
Pulmonary medications	1.00 (0.94-1.05)	0.93 (0.85-1.03)	1.03 (0.97-1.11)	**0.68 (0.48-0.97)**
Vasopressors	**1.81 (1.70-1.93)**	**3.00 (2.49-3.61)**	**1.66 (1.55-1.79)**	**1.52 (1.12-2.04)**
Mechanical ventilation				
Invasive	**1.08 (1.01-1.16)**	**2.48 (1.98-3.10)**	1.02 (0.95-1.10)	0.92 (0.65-1.29)
Noninvasive	**1.16 (1.06-1.27)**	**2.03 (1.60-2.57)**	1.03 (0.94-1.13)	1.22 (0.80-1.86)
Organ failure				
Respiratory	**2.48 (2.34-2.63)**	**3.84 (3.45-4.29)**	**2.03 (1.90-2.18)**	**1.47 (1.06-2.04)**
Cardiovascular	**1.91 (1.80-2.02)**	**1.90 (1.68-2.15)**	**1.81 (1.69-1.94)**	**1.36 (1.02-1.82)**
Neurological	**1.53 (1.38-1.71)**	**1.64 (1.33-2.02)**	**1.40 (1.23-1.58)**	**1.92 (1.03-3.65)**
Renal	**1.58 (1.51-1.66)**	**1.77 (1.62-1.94)**	**1.46 (1.38-1.55)**	1.15 (0.87-1.51)
Hematologic	**1.65 (1.52-1.80)**	**1.97 (1.67-2.32)**	**1.53 (1.39-1.69)**	1.18 (0.73-1.90)
Hepatic	**2.54 (2.08-3.11)**	**3.21 (2.05-5.00)**	**2.38 (1.90-2.99)**	0.66 (0.20-2.16)
Antibiotic therapy				
GC-CAP therapy	**0.67 (0.63-0.70)**	**0.60 (0.54-0.67)**	**0.73 (0.69-0.79)**	0.73 (0.50-1.08)
Pseudomonal therapy	**1.25 (1.19-1.32)**	**1.68 (1.53-1.85)**	1.05 (0.99-1.12)	0.76 (0.54-1.06)
Atypical therapy	**0.45 (0.43-0.48)**	**0.59 (0.53-0.66)**	**0.46 (0.43-0.49)**	0.75 (0.52-1.08)
MRSA culture positivity	**0.66 (0.59-0.74)**	**0.58 (0.45-0.73)**	**0.66 (0.58-0.75)**	**0.56 (0.31-0.99)**

Bold indicates statistical significance; *MRSA* Methicillin-resistant *Staphylococcus aureus*; Race was ordered as black versus nonblack; *COPD* Chronic obstructive pulmonary disease, *GC-CAP* Guideline-concordant community-acquired pneumonia

## Discussion

CO-MRSA pneumonia is an evolving problem, and clinicians need strategies to determine appropriate candidates for empiric MRSA therapy. Shorr *et al.* developed a risk score to specifically identify CO-pneumonia patients at risk for MRSA infection [14]. Using a MRSA risk score similar to Shorr *et al.*, our study demonstrates a survival advantage for CO-pneumonia among high-risk patients who received initial MRSA therapy. The number needed to treat with initial MRSA therapy to save one life in the high-risk group was 5. This survival advantage was not present for patients in the low and medium-risk groups.

Our study also supports the notion that the MRSA risk score might be a better alternative to guide empiric MRSA therapy in patients with CO-pneumonia as compared to the HCAP criteria. Several studies have demonstrated that the HCAP criteria have low specificity for MRSA pneumonia, and that grouping risk factors for MRSA with other gram-negative MDR pathogens may lead to inappropriate treatment [11, 22–25]. Recently, Chalmers *et al.* conducted a meta-analysis of 24 studies that compared HCAP and CAP cohorts. The study concluded that the ability for the HCAP criteria to appropriately identify patients with MDR pathogens (including MRSA) was low and did not meet the threshold for clinical use [26]. In addition, most studies evaluating the utility of HCAP criteria to guide broad-spectrum therapy, including MRSA therapy, have not demonstrated improved mortality with guideline-concordant therapy [12, 13]. In a large Canadian cohort study, Grenier *et al.* were unable to demonstrate a 30-day mortality benefit in HCAP patients treated with guideline-concordant antimicrobials [13]. In 2012, Madaras-Kelly *et al.* evaluated guideline-endorsed HCAP regimens by stratifying patients based on risk for MDR pathogens [27]. This study demonstrated that patients who were at higher risk for MDR pathogens had a survival benefit if they were treated with guideline-endorsed therapies; however, those with low risk for MDR pathogens who received guideline-endorsed therapies had significantly higher mortality.

One advantage of the MRSA risk score is weighting of important risk factors. The MRSA risk score assigns more weight (2 points each) to patients with recent hospitalization and severe pneumonia requiring ICU admission compared to the other risk factors (1 point each). In a recent study stratifying risk factors for MDR pathogens in CO-pneumonia patients, recent hospitalization and severe pneumonia were found to be independent predictors of mortality [28]. This highlights a key feature of the MRSA risk score, because patients with either of those two risk factors cannot be classified into the low-risk group.

Recently, Minejima *et al.* compared a smaller cohort of CO-pneumonia patients with culture-proven MRSA pneumonia and non-MRSA pneumonia. They found 37 % of patients with non-MRSA pneumonia met HCAP criteria and 28 % received unnecessary MRSA therapy. Had the MRSA risk score been applied, unwarranted MRSA therapy could have been decreased by 20 % in low-risk patients without MRSA pneumonia [29]. Our findings further support the idea that the new MRSA risk score can help identify low-risk CO-pneumonia patients who are unlikely to benefit from empiric MRSA therapy.

Our study has important limitations. First, prior studies have identified risk factors associated with CO-MRSA pneumonia that were not included in the MRSA risk score. These include: MRSA infection in the past year, known MRSA colonization, necrotizing or cavitary pneumonia, severity-of-illness scores, and preceding or concurrent influenza [19, 29–36]. Similarly, there are patient and provider characteristics that were not included in our analyses that might affect patient outcomes. These include specific antimicrobial medications received, non-antimicrobial medications, pathogens, antimicrobial susceptibilities, patient functional status and clinical presentation, and provider preferences. The study inclusion dates might not provide a good reflection of current prescribing practices for CO-pneumonia; however it is important to note that we sorted the patients into guideline-concordant and discordant groups according to the recommendations in the most recent CAP guidelines. We evaluated the impact of guideline-concordant therapy, pseudomonal therapy, and atypical therapy in our multivariable models; however, we did not study specific medications and combinations, some of which might be associated with better outcomes. The data source included a predominately elderly male population. This population had higher 30-day mortality than that typically seen in non-VA populations, which could limit the generalizability of our results. In addition, the older age of the population precluded our ability to assess Shorr’s MRSA risk criteria for age <30 years. Other changes in our variable definitions might also have affected outcomes. We altered the prior IV antibiotic use criteria to include use in the prior 90 days, rather than 30 days. This is likely to result in a larger proportion of patients who meet this criterion, and might bias the population to higher MRSA risk scores.

ICD-9-CM codes were used to identify pneumonia patients, pathogens, and baseline characteristics. This approach could potentially lead to misclassification bias, or underestimate the true prevalence of the pathogens, and cannot be considered equivalent to a medical chart review. There was no MRSA pneumonia code during the time these patients were treated for pneumonia; therefore, MRSA pneumonia was defined as a patient with a pneumonia ICD-9 code plus a general MRSA ICD-9 code that was not specific for MRSA pneumonia. Because of this, the general MRSA code could represent some other infectious process occurring simultaneously with pneumonia and not necessarily MRSA pneumonia. Furthermore, the V09.0 code is an imprecise way to identify MRSA infections, in general [37]. However, there is evidence to suggest that the code more accurately identifies MRSA infections in community-onset patients (like the ones in this study) than in hospital-onset patients [38]. In the absence of a single code that couples disease with pathogen, many large databases, like ours, rely on existing ICD9 codes, like the V09.0 MRSA code plus pneumonia codes, for case definitions. A published study from another group [39], and one from our own group [40], demonstrate examples where investigators have used the V09.0 code to identify patients with MRSA infections.

Also, it is possible that patients in the MRSA low-risk group did worse because of the greater prevalence of some other problem pathogen, for which anti-MRSA antibiotics have no activity. Reliance on ICD-9 codes for pathogen identification resulted in a lower rate of pathogen identification, so this is not the best study design to identify problem pathogens. Since this is an observational study, the impact of MRSA therapy would be better assessed if the propensity score of MRSA therapy was adjusted for in the analysis, so that the bias due to observed confounding (baseline covariates that were associated with MRSA use) could be minimized. Also, for unobserved confounding, an instrumental variable approach would further reduce the bias due to unobserved confounding. However, our analyses did not consider the propensity score nor instrumental variable adjustments due to the limited baseline data available from the electronic medical record database and lack of a credible instrumental variable that can be identified in the literature.

## Conclusions

This study demonstrated improved survival with initial MRSA therapy in high-risk CO-pneumonia patients. The MRSA risk score might help spare MRSA therapy for only those patients who are likely to benefit.

## Additional files

Additional file 1:
**ICD-9-CM codes for patient variables and bacterial pathogens.** (DOCX 25 kb) Additional file 2:
**List of medications used within 90 days of admission, by class.** (DOCX 24 kb)

## Abbreviations

CO: Community-onset
MRSA: Methicillin-resistant *Staphylococcus aureus*
CAP: Community-acquired pneumonia
HCAP: Healthcare-associated pneumonia
MDR: Multidrug-resistant
VHA: Veterans Health Administration
ICD-9-CM: International Classification of Diseases, Ninth Revision, Clinical Modification codes
ICU: Intensive care unit
IV: Intravenous
GC: Guideline-concordant
aOR: Adjusted odds ratio
CI: Confidence interval
COPD: Chronic obstructive pulmonary disease
IQR: Interquartile range
