# Supplementary material for: Application of a methicillin-resistant Staphylococcus aureus risk score for community-onset pneumonia patients and outcomes with initial treatment
Source: BMC Infect Dis. 2015 Sep 18;15:380. doi: 10.1186/s12879-015-1119-1 (PMC4575496; doi:10.1186/s12879-015-1119-1)
Supplement: Additional file 1: — ICD-9-CM codes for patient variables and bacterial pathogens. (DOCX 25 kb) [file 12879_2015_1119_MOESM1_ESM.docx]

**Appendix 1.** ICD-9-CM codes for patient variables and bacterial pathogens

| **Variable** | **ICD-9-CM Codes** |
| --- | --- |
| Hemodialysis | 403.01, 403.11, 403.91, 404.02, 404.12, 404.92, 404.93, 584, 585, 586 |
| Invasive mechanical ventilation | 96.7 |
| Non-invasive mechanical ventilation | 93.90, 93.91 |
| Comorbid conditions | |
| Dementia | 290 |
| Myocardial infarction | 410, 412 |
| Congestive heart failure | 428 |
| Cerebrovascular disease | 430-438 |
| COPD | 490-496, 500-505, 506.4 |
| Liver disease | 571.2, 571.4, 571.5, 571.6, 572.2-572.8, 456.0-456.21 |
| Diabetes | 250.0-250.3, 250.4, 250.5, 250.6, 250.7, 250.8, 250.9 |
| Renal disease | 582, 583, 585, 586, 588 |
| Neoplastic disease | 140-172, 174-208 |
| HIV/AIDS | 42-44, V08 |
| Organ failure | |
| Respiratory | 518.81, 518.82, 518.85, 518.89, 786.09, 799.1, 96.7 |
| Cardiovascular | 458.0, 458.8, 458.9, 785.5, 796.3 |
| Neurologic | 293, 348, 348.3, 780.01, 780.09, 89.14 |
| Renal | 580, 584, 585, 039.95 |
| Hematologic | 286.2, 286.6, 286.9, 287.3, 287.4, 287.5 |
| Hepatic | 570, 572.2, 573.3 |
| Bacterial Pneumonia Pathogens | |
| *Streptococcus pneumoniae* | 481 |
| *Streptococcus*, other | 482.30, 482.31, 482.32, 482.40 |
| *Staphylococcus*, unspecified | 482.40 |
| *Staphylococcus aureus* | 482.41 |
| MRSA | V09.0 |
| *Klebsiella pneumoniae* | 482.0 |
| *Pseudomonas* spp. | 482.1 |
| *Haemophilus influenzae* | 482.2 |
| *Escherichia coli* | 482.82 |
| Other gram-negative bacteria | 482.83 |
| *Legionella* spp. | 482.84 |
| *Mycoplasma pneumoniae* | 483.0 |
| *Chlamydia* spp. | 483.1 |
| Anaerobes | 482.81 |

COPD: chronic obstructive pulmonary disease; HIV/AIDS: human immunodeficiency virus/acquired immunodeficiency syndrome; MRSA: methicillin-resistant *Staphylococcus aureus*
