# Supplementary material for: Application of a methicillin-resistant Staphylococcus aureus risk score for community-onset pneumonia patients and outcomes with initial treatment
Source: BMC Infect Dis. 2015 Sep 18;15:380. doi: 10.1186/s12879-015-1119-1 (PMC4575496; doi:10.1186/s12879-015-1119-1)
Supplement: Additional file 2: — List of medications used within 90 days of admission, by class. (DOCX 24 kb) [file 12879_2015_1119_MOESM2_ESM.docx]

**Appendix 2.** List of medications used within 90 days of admission, by class

| **Drug Class** | **Medications** |
| --- | --- |
| Cardiovascular medications | Aliskiren, ambrisentan, amiloride, amlodipine, atenolol, atorvastatin, benazepril, bendroflumethizide, bepridil, betaxolol, bisoprolol, bretylium, bumetanide, carteolol, carvedilol, chlorothiazide, chlorthalidone, clevidipine, clonidine, diazoxide, digoxin, diltiazem, disopyramide, dofetilide, doxazosin, eplerenone, epoprostenol, esmolol, ethacrynic acid, felodipine, fenoldopam, flecainide, furosemide, guanabenz, guanadrel, guanethidine, guanfacine, hydralazine, hydrochlorothiazide, ibutilide, iloprost, inamrinone, indapamide, isosorbide, isoxsuprine, isradipine, labetalol, mannitol, mecamylamine, methyclothiazide, methyldopa, metolazone, metoprolol, metyrosine, mexiletine, milrinone, minoxidil, moricizine, nadolol, nebivolol, nesiritide, nicardipine, nifedipine, nimodipine, nisoldipine, nitroglycerin, papaverine, penbutolol, perindopril, phentolamine, pindolol, polythiazide, prazosin, procainamide, propafenone, propranolol, quinidine, reserpine, sotalol, spironolactone, terazosin, timolol, tocainidine, tolazoline, torsemide, treprostinil, triamterene, trimethaphan, verapamil |
| Antidiabetic medications | Acarbose, chlorpropamide, exenatide, glimepiride, glipizide, glyburide, insulin, metformin, miglitol, nateglinide, pioglitazone, pramlintide, repaglinide, rosiglitazone, sitagliptin, tolazamide, tolbutamide |
| Inhaled corticosteroids | Flunisolide, fluticasone, mometasone, triamcinolone |
| Systemic corticosteroids | Betamethasone, budesonide, cortisone, dexamethasone, hydrocortisone, methylprednisolone, prednisolone, prednisone, triamcinolone |
| Pulmonary medications | Acetylcysteine, albuterol, aminophylline, arformoterol, beractant, bitolterol, caffeine, calfactant, cetyl alcohol, ciclesonide, colfosceril, cromolyn, dornase alfa, doxapram, formoterol, ipratropium, levalbuterol, metaproterenol, montelukast, nedocromil, nitric oxide, omalizumab, pirbuterol, poractant alfa, salmeterol, terbutaline, theophylline, tobramycin, tyloxapol, zafirlukast, zileuton |
